# Deep Molecular Characterization of HIV-1 Dynamics under Suppressive HAART

**DOI:** 10.1371/journal.ppat.1002314

**Published:** 2011-10-27

**Authors:** Maria J. Buzón, Francisco M. Codoñer, Simon D. W. Frost, Christian Pou, Maria C. Puertas, Marta Massanella, Judith Dalmau, Josep M. Llibre, Mario Stevenson, Julià Blanco, Bonaventura Clotet, Roger Paredes, Javier Martinez-Picado

**Affiliations:** 1 Institut de Recerca de la SIDA, IrsiCaixa, Hospital Universitari Germans Trias i Pujol, Universitat Autònoma de Barcelona, Badalona, Spain; 2 Department of Veterinary Medicine, University of Cambridge, Cambridge, United Kingdom; 3 Unitat VIH, Hospital Universitari Germans Trias i Pujol, Universitat Autònoma de Barcelona, Badalona, Spain; 4 University of Miami Miller School of Medicine, Miami, Florida, United States of America; 5 Institució Catalana de Recerca i Estudis Avançats (ICREA), Barcelona, Spain; NIH/NIAID, United States of America

## Abstract

In order to design strategies for eradication of HIV-1 from infected individuals, detailed insight into the HIV-1 reservoirs that persist in patients on suppressive antiretroviral therapy (ART) is required. In this regard, most studies have focused on integrated (proviral) HIV-1 DNA forms in cells circulating in blood. However, the majority of proviral DNA is replication-defective and archival, and as such, has limited ability to reveal the dynamics of the viral population that persists in patients on suppressive ART. In contrast, extrachromosomal (episomal) viral DNA is labile and as a consequence is a better surrogate for recent infection events and is able to inform on the extent to which residual replication contributes to viral reservoir maintenance. To gain insight into the diversity and compartmentalization of HIV-1 under suppressive ART, we extensively analyzed longitudinal peripheral blood mononuclear cells (PBMC) samples by deep sequencing of episomal and integrated HIV-1 DNA from patients undergoing raltegravir intensification. Reverse-transcriptase genes selectively amplified from episomal and proviral HIV-1 DNA were analyzed by deep sequencing 0, 2, 4, 12, 24 and 48 weeks after raltegravir intensification. We used maximum likelihood phylogenies and statistical tests (AMOVA and Slatkin-Maddison (SM)) in order to determine molecular compartmentalization. We observed low molecular variance (mean variability ≤0.042). Although phylogenies showed that both DNA forms were intermingled within the phylogenetic tree, we found a statistically significant compartmentalization between episomal and proviral DNA samples (*P*<10^−6^ AMOVA test; *P* = 0.001 SM test), suggesting that they belong to different viral populations. In addition, longitudinal analysis of episomal and proviral DNA by phylogeny and AMOVA showed signs of non-chronological temporal compartmentalization (all comparisons *P*<10^−6^) suggesting that episomal and proviral DNA forms originated from different anatomical compartments. Collectively, this suggests the presence of a chronic viral reservoir in which there is stochastic release of infectious virus and in which there are limited rounds of *de novo* infection. This could be explained by the existence of different reservoirs with unique pharmacological accessibility properties, which will require strategies that improve drug penetration/retention within these reservoirs in order to minimise maintenance of the viral reservoir by de novo infection.

## Author Summary

In the majority of HIV-1 positive patients, antiretroviral therapy (ART) effects a sustained reduction in plasma viremia to below detectable levels. Despite this, replication competent viruses persist and fuel viremia if antiretroviral treatment is interrupted. This viral persistence stands in the way of viral eradication through ART. While this ability to persist in the face of therapy is generally considered to be attributable to a reservoir of latently infected cells, there is debate as to how this reservoir is maintained and in particular, whether there is replenishment of the reservoir by low level, residual replication. Novel antiviral agents targeting the viral integrase offer tools to explore the viral reservoirs that persist in the face of ART and we have shown that raltegravir perturbs these reservoirs as evidenced by an accumulation of episomal DNA upon rategravir intensification (Buzon et al., 2010). Through “deep sequencing” technology, we have longitudinally analyzed the genotypes of HIV episomes and integrated HIV DNA to evaluate whether they represent interrelated sequences or whether they have distinct origins. Statistical methods showed molecular compartmentalization, among and within episomal and integrated HIV-1 DNA samples, and suggest that episomal DNA in PBMC originates from a cellular/anatomic reservoir that is not revealed by sequencing of proviral DNA in PBMC in this study. These, and other data, suggest that ongoing replication, which can be blocked by adding raltegravir, occurs from proviruses that are genetically distinguishable from those detected at >1% frequency in these circulating blood cells.

## Introduction

In the majority of HIV-1 infected individuals antiretroviral therapy (ART) is able to sustain suppression of plasma viral load to undetectable levels (<50 copies HIV RNA/ml plasma) for sustained intervals. However, viremia resumes if treatment is interrupted. Therefore, HIV-1 is able to persist in the face of suppressive ART. In addition low-level residual viremia has been detected with ultrasensitive assays that are able to measure down to several copies of HIV RNA/ml plasma [1], [2]. It has been suggested that low level viremia in ART-suppressed patients represents release of viral particles by long-lived latently infected CD4+ T-cells [3], [4], [5] or virions produced as a result of low-level, residual viral replication [6], [7], [8], [9], [10], [11]. The nature of this residual viremia, remains poorly understood, mainly because the very low number of virions in plasma limits its molecular characterization [3], [12], [13].

Intensification protocols employing integrase inhibitors have been used to probe the viral reservoirs that persist in ART-suppressed patients. When viral integration is inhibited, the linear viral genome, which is the precursor to the integrated provirus, is converted to episomes [14], [15]. Although sequences gleaned from episomal DNAs could be present in both productive and non-productive infections, integrase inhibition specifically results in increased episome formation and since linear cDNA is a product of reverse transcription, increases in episomal cDNA in blood cells after starting raltegravir indicates *de novo* infection and blocked integration. Because of the dynamic nature of episomes, they harbor a higher percentage of contemporary sequences as compared to proviral sequences that contain a higher percentage of archival sequences. Therefore, although episomes are dead-end products of viral replication, sequences contained within them will also be observed in functional viral genomes. As a consequence, characterization of the nature of episomal HIV-1 DNA during raltegravir intensification of a suppressive HAART regimen could provide new insights into the molecular diversity and compartmentalization of the viral reservoirs that persist in the face of suppressive therapy. We previously reported that raltegravir intensification of HAART-suppressed patients affected HIV-1 replication and immune dynamics in a large percentage of these patients [16], [17]. In order to gain further insight into the molecular diversity, population structure, and compartmentalization of replicative viral forms under suppressive HAART, episomal and integrated HIV-1 DNA samples were longitudinally analyzed by deep sequencing after intensification with raltegravir. We found signs of molecular compartmentalization distinguishing episomal and integrated HIV-1 DNA populations in PBMC, suggesting that proviruses in a cellular/anatomical compartment other than those cells may give rise to stochastic release of replication-competent virus during HAART.

## Results

### Deep sequencing

The study sample comprised two participants from our previously reported raltegravir-intensification study [16], [17] who had plasma viremia below 50 HIV-1 RNA copies per ml for two years on stable HAART. Subjects were selected based on sample availability. Reverse-transcriptase genes from episomal and integrated HIV-1 DNA were specifically amplified and analyzed at weeks 0, 2, 4, 12, 24 and 48 following raltegravir intensification. Only viral sequences present in ≥1% of the virus population were considered for further analysis. The median number and interquartile range of episomal HIV-1 DNA clonal sequences for patients 1 and 2 was 3,063 (2,017–3,473) and 4,040 (2,106–6,131), respectively. For integrated HIV-1 DNA in patients 1 and 2, the median was 2,645 (1,570–3,592) and 2,889 (2,247–4,184), respectively.

### Episomal and integrated HIV-1 DNA belong to different viral populations

**Figure 1:**
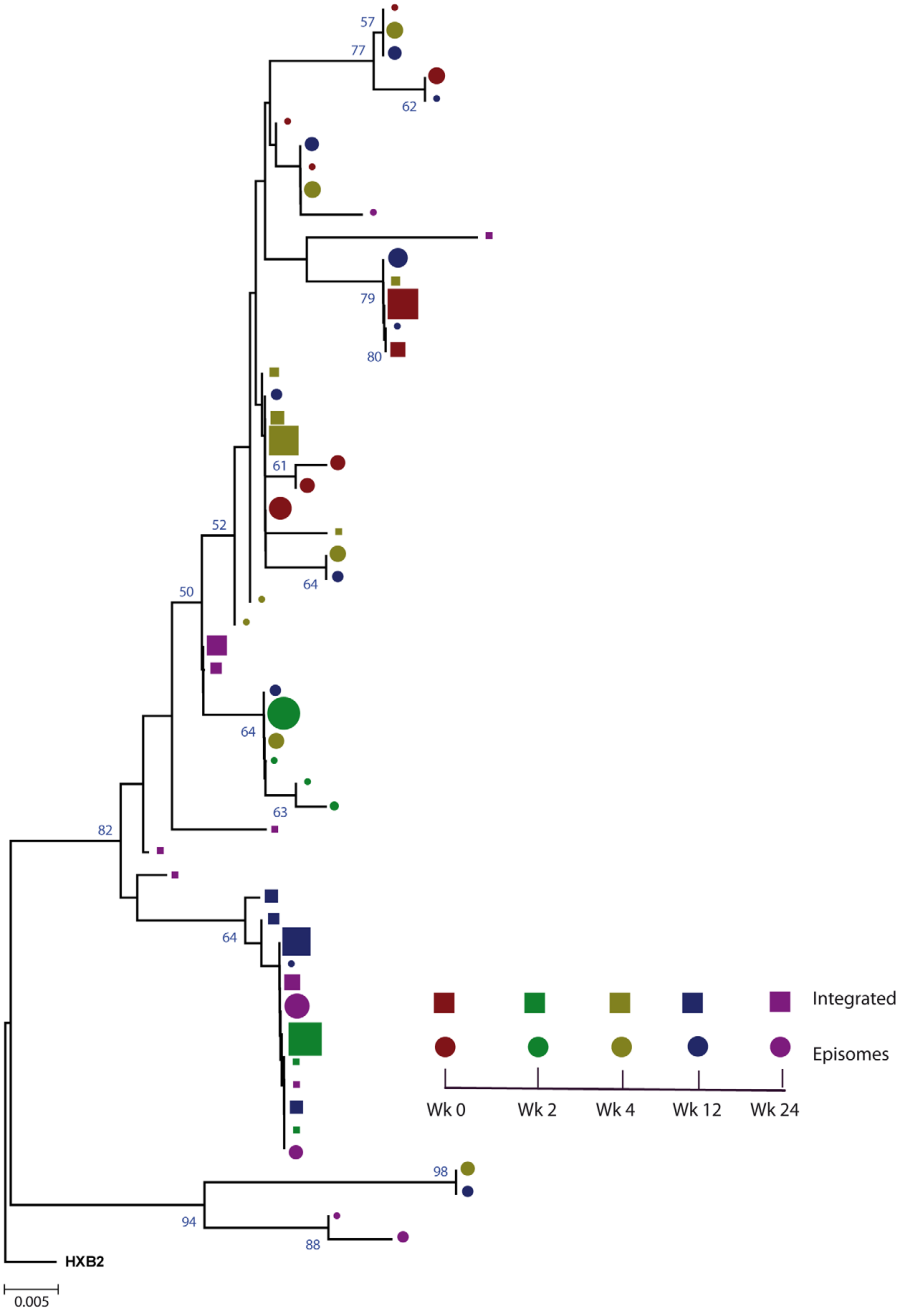
Phylogenetic tree of patient 1. A neighbor-joining approach, as implemented in MEGA4, was used to construct a phylogenetic tree with the best evolutionary model found in jModeltest v0.1.1. Circles and squares represent longitudinal episomal and integrated DNA sequences, respectively. Legends of phylogenetic trees represent weeks available for further analysis. Sizes of the symbols represent the different percentages of clonal sequences. 1,000 bootstrap replicates were performed; only values greater than 50% are shown at tree nodes.

**Figure 2:**
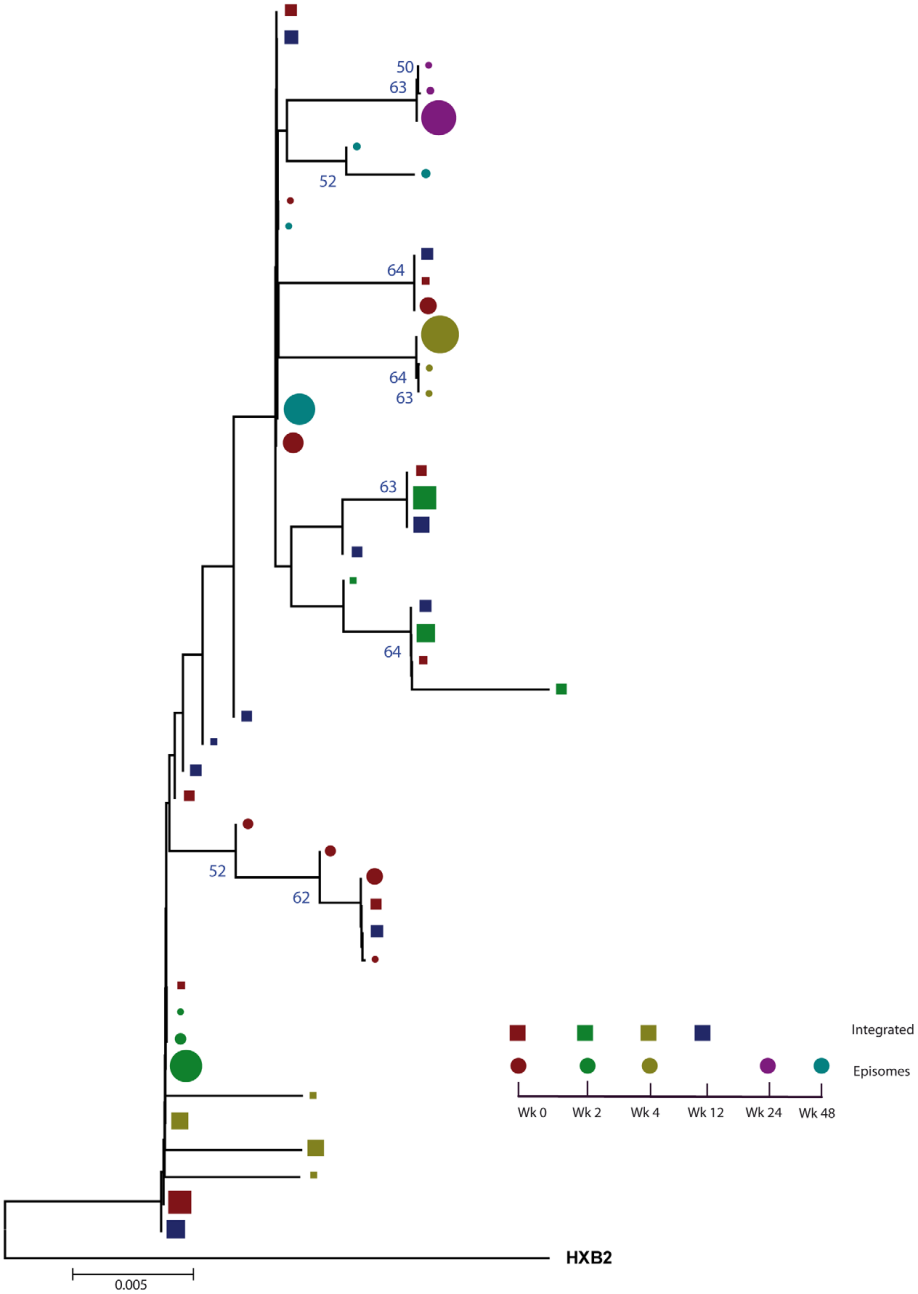
Phylogenetic tree of patient 2. A neighbor-joining approach, as implemented in MEGA4, was used to construct a phylogenetic tree with the best evolutionary model found in jModeltest v0.1.1. Circles and squares represent longitudinal episomal and integrated DNA sequences, respectively. Legends of phylogenetic trees represents weeks available for further analysis. Size of the symbols represent the different percentages of clonal sequences. 1,000 bootstrap replicates were performed; only values greater than 50% are shown at tree nodes.

We constructed a phylogenetic tree for each patient to assess if episomal and integrated HIV-1 DNA sequences belonged to different genetic populations or to one intermixed population. We used a neighbor-joining approach, as implemented in MEGA4 [18], to construct a phylogenetic tree for each patient with the best evolutionary model found in jModeltest v0.1.1. Phylogenetic trees did not show a clear cluster differentiation of both DNA forms (Figs. 1 and 2), suggesting a lack of population structure between both DNA samples, at least at the sequence composition level. Even when sequences do not clearly group into separate branches, statistical analysis can still reveal differences in sequence diversity between different HIV populations [12], [19], [20]. Therefore, to better assess possible compartmentalization, we performed a population structure test based on the analysis of molecular variance (AMOVA) on pairwise genetic distances and percentages of the presence of each clone in each population [21]. This test showed different ratios of population structure (FST) between episomal and integrated sequences (Table 1), which all were statistically significant (*P*<10^−6^) in both patients. As different tests of population structure can yield contradictory results, we performed a recommended conservative analysis [22] using a complementary compartmentalization test. We applied the Slatkin-Maddison test [23], which is based only on tree topology comparison. Consistent with the AMOVA test, the results of the Slatkin-Maddison analysis showed only a few migration events between episomal and integrated samples: 12 out of 55 possible migration events, and 9 out of 46 possible migration events in patients 1 and 2 respectively (*P* = 0.001) (Table 1). In addition, we obtained similar results when a longitudinal point-by-point comparison was performed between both DNA viral forms, except for three samples where the migration events had a high probability of being random (Table 1). Interestingly, two of these three samples were taken at the study baseline, i.e. right before HAART intensification with raltegravir. Overall, these results point to statistically significant compartmentalization between episomal and integrated DNA samples and suggest that they belong to different viral populations.

**Table 1:** Population structure analysis.

		*Mean Diversity*		AMOVA		Slatkin-Maddison Test	
Patient	Time	*Π_Epi_*	*Π_Int_*	*F_ST_*	*P-value*	Migrations	*Prob* *
1	W0	0.0091	<0.0001	0.7287	<10^−6^	1	0.105
	W2	0.0019	<0.0001	0.9889	<10^−6^	1	0.056
	W4	0.0265	0.0063	0.1891	<10^−6^	2	0.026
	W12	0.0313	0.0019	0.8412	<10^−6^	2	0.033
	W24	0.0422	0.0198	0.5160	<10^−6^	4	0.578
	Total	0.0278	0.0201	0.1684	<10^−6^	12	0.001
2	W0	0.0057	0.0081	0.1679	<10^−6^	5	0.584
	W2	<0.0001	0.0067	0.1092	<10^−6^	1	0.058
	W4	<0.0001	0.0084	0.7680	<10^−6^	1	0.057
	W12	NA	0.0054	NA	NA	NA	NA
	W24	<0.0001	NA	NA	NA	NA	NA
	W48	0.0019	NA	NA	NA	NA	NA
	Total	0.0076	0.0079	0.1457	<10^−6^	9	0.001

Mean internal diversity for each time point and DNA source is shown for each patient. Genetic diversity (π), defined as the average number of nucleotide differences per site between any two sequences chosen randomly from the sample population, is calculated with the best evolutionary model found in the jmodeltest Tamura and Nei model [45]. The fixation index, *F_ST,_* from the AMOVA analysis is a measure of the diversity of randomly chosen sequences within the same sub-population relative to that found in the entire population. A zero value implies that the two populations mix freely, while a value of one implies that the two subpopulations are completely separate. The Slatkin Maddison test uses an estimate of the number of ‘migrations’ between subpopulations to assess population structure, with smaller numbers of migrations (for a given number of sequences) indicating more structure. Both AMOVA and the Slatkin-Maddison test calculate a p value for the null hypothesis of no population structure by randomly permuting sequences between subpopulations; they differ as the AMOVA takes into account the frequency of each unique sequence or haplotype, whereas the Slatkin Maddison test does not.

*Probability that a migration event is random after 10,000 resampling replicates.

NA, indicates that the test or the data is not applicable or available.

### Non-chronological temporal compartmentalization within episomal and integrated HIV-1 viral forms

**Figure 3:**
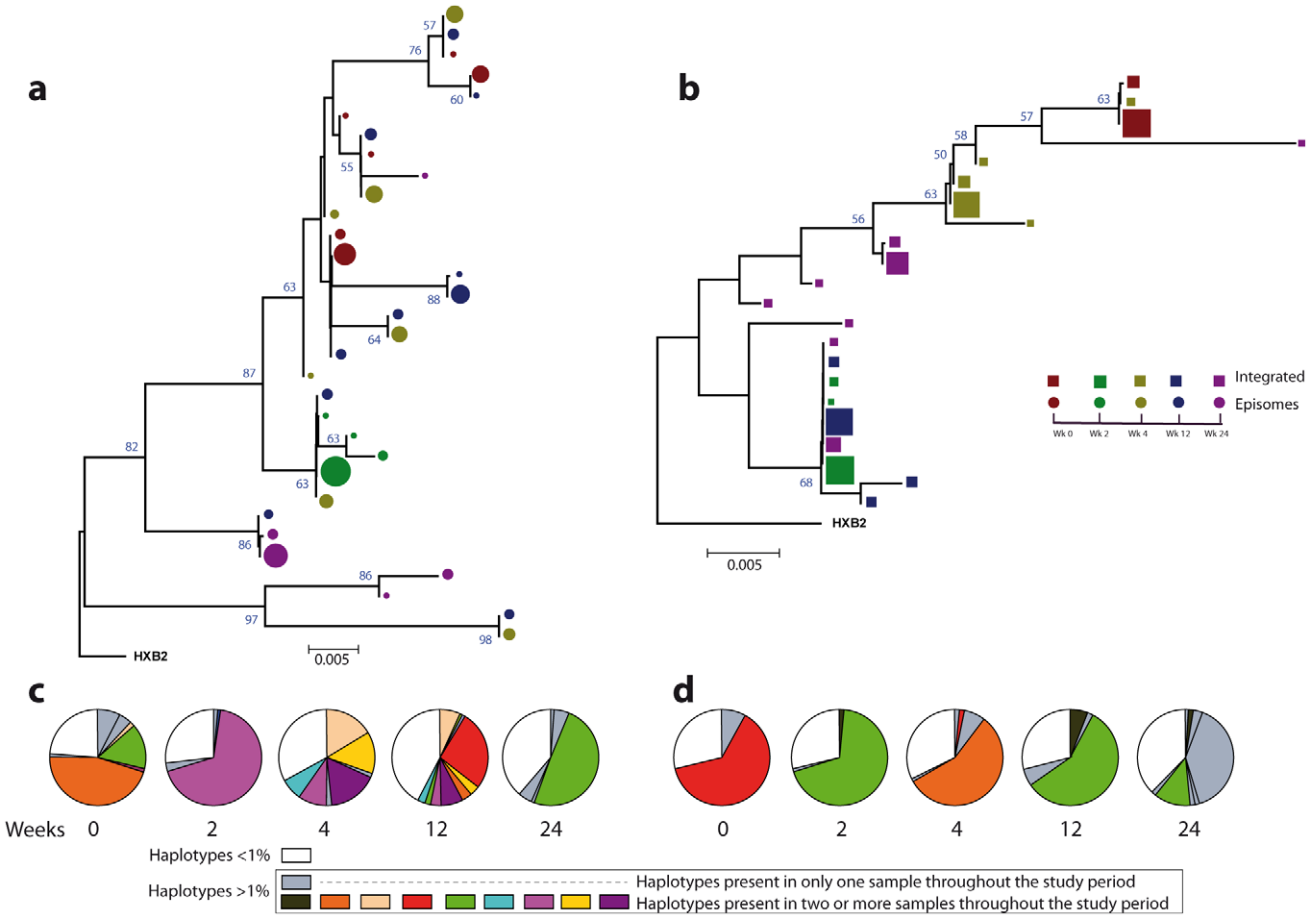
Phylogenetic tree and proportions of episomal and integrated HIV-1 sequences in patient 1. A neighbor-joining approach, as implemented in MEGA4, was used to construct a phylogenetic tree with the best evolutionary model found in jModeltest v0.1.1. **a.** Circles represent longitudinal episomal DNA sequences. **b.** Squares represent longitudinal integrated DNA sequences. Legend of phylogenetic trees represents weeks available for further analysis. Sizes of the symbols represent the different percentages of clonal sequences. **c.** Longitudinal representation of the clonal variability of each episomal sample. **d.** Longitudinal representation of the clonal variability of each integrated sample. Areas of pie charts in white shading indicate sequences present with a frequency below 1%; gray shading indicates sequences with a frequency above 1% present in only one sample throughout the study period; colors represent sequences with a frequency above 1% present in two or more samples throughout the study period. 1,000 bootstrap replicates were performed; only values greater than 50% are shown at tree nodes.

**Figure 4:**
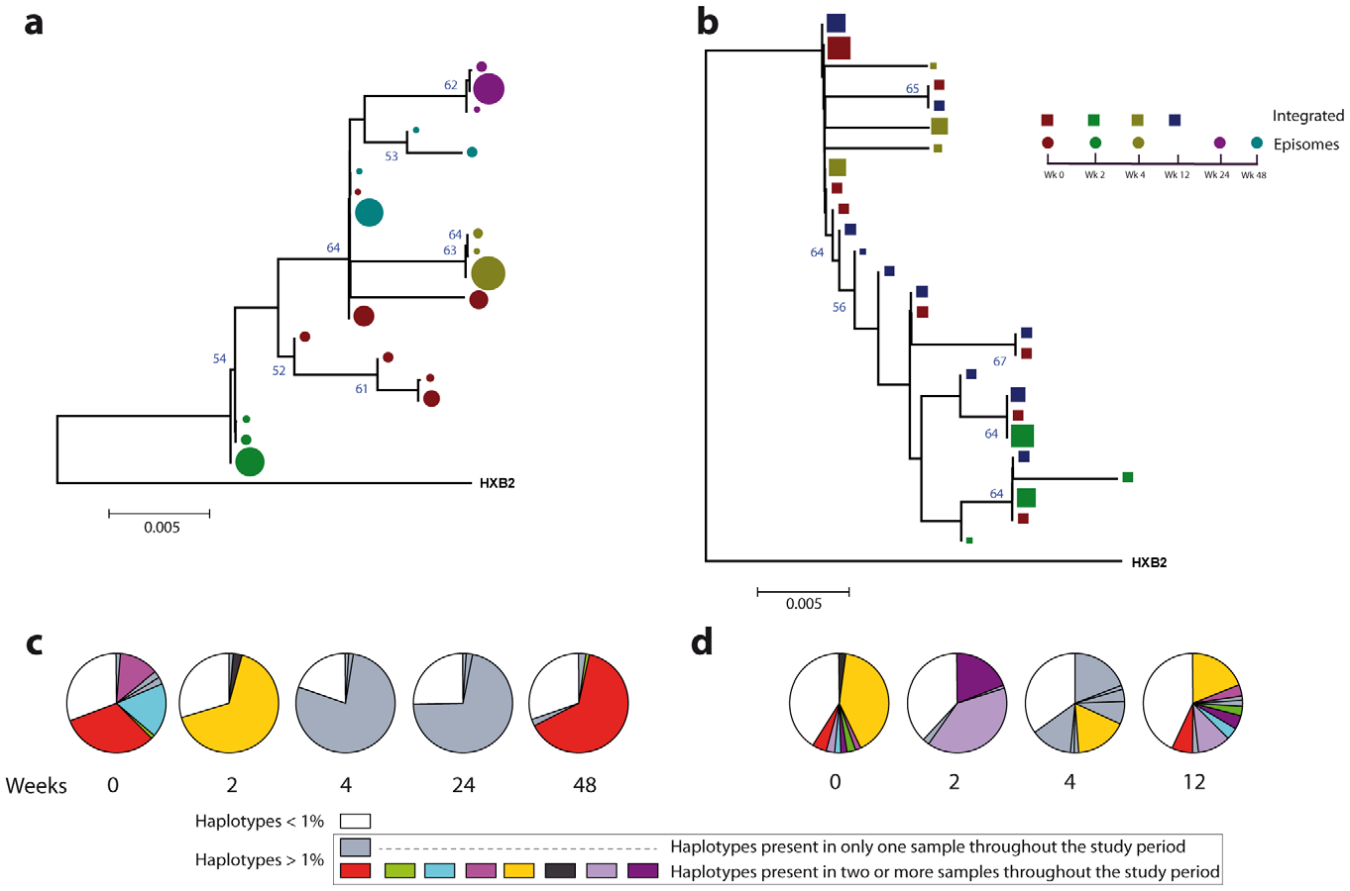
Phylogenetic tree and proportions of episomal and integrated HIV-1 sequences in patient 2. A neighbor-joining approach as implemented in MEGA4 was used to construct a phylogenetic tree with the best evolutionary model found in jModeltest v0.1.1. **a.** Circles represent longitudinal episomal DNA sequences. **b.** Squares represent longitudinal integrated DNA sequences. Legend of phylogenetic trees represents weeks available for further analysis. Sizes of the symbols represent different percentage of clonal sequences. **c.** Longitudinal representation of clonal variability of each episomal sample. **d.** Longitudinal representation of clonal variability of each integrated sample. Areas of pie charts in white shading indicate sequences present with a frequency below 1%; gray shading indicates sequences with a frequency above 1% present in only one sample throughout the study period; colors represent sequences with a frequency above 1% present in two or more samples throughout the study period. 1,000 bootstrap replicates were performed; only values greater than 50% are shown at tree nodes.

We next assessed whether longitudinal episomal and integrated DNA sequences had a temporal structure. Firstly, we constructed a separate neighbor-joining phylogenetic tree for each patient and each viral DNA form (Fig. 3 and 4). Phylogenies showed evidence of a temporal structure within episomal and integrated viral DNA forms across different time-points. Temporal structure was more evident in the episomal samples of patient 2 (Fig. 4a) and the integrated samples of patient 1 (Fig. 3b). However, a clear sign of temporal structure was difficult to observe in the remaining phylogenetic trees (Fig. 3a and 4b). Therefore, we used the AMOVA and Slatkin-Maddison tests to again assess the presence of temporal population structure within each viral DNA form. AMOVA showed that all longitudinal comparisons within episomal and integrated samples were significantly different (*P*<10^−6^) indicating different temporal population structures (Tables 2–5). Statistically significant Slatkin-Maddison results were partly consistent with those detected by AMOVA (Tables 2–5). Discrepancies between both tests might be due to the large number of sequences with the same haplotype (sometimes present in both compartments). In fact, the performance of the Slatkin-Maddison test might be limited when there is a combination of relatively short sequence, high depth and low within-patient diversity [23], as in this case.

**Table 2:** Temporal population structure: AMOVA test for comparison between longitudinal episomal HIV-1 DNA.

	AMOVA test - Episomal HIV-1 DNA					
	F_st_ (*P*-value)					
	W0	W2	W4	W12	W24	W48
**W0**	–	*0.787 (<10^−6^)*	*0.107 (<10^−6^)*	*0.238 (<10^−6^)*	*0.764 (<10^−6^)*	*NA*
**W2**	**0.584 (<10^−6^)**	–	*0.616 (<10^−6^)*	*0.762 (<10^−6^)*	*0.873 (<10^−6^)*	*NA*
**W4**	**0.664 (<10^−6^)**	**0.998 (<10^−6^)**	–	*0.133 (<10^−6^)*	*0.592 (<10^−6^)*	*NA*
**W12**	**NA**	**NA**	**NA**	–	*0.655 (<10^−6^)*	*NA*
**W24**	**0.669 (<10^−6^)**	**0.997 (<10^−6^)**	**0.989 (<10^−6^)**	**NA**	–	*NA*
**W48**	**0.273 (<10^−6^)**	**0.969 (<10^−6^)**	**0.964 (<10^−6^)**	**NA**	**0.965 (<10^−6^)**	–

The longitudinal comparisons of patient 1 are indicated in the upper-right hemi-matrix of the table (*italics*); those of patient 2 are shown in the lower-left hemi-matrix of the table (**bold**).

**Table 3 ppat-1002314-t003:** Temporal population structure: AMOVA test for comparison between longitudinal integrated HIV-1 DNA.

	AMOVA test - Integrated HIV-1 DNA					
	F_st_ (*P*-value)					
	W0	W2	W4	W12	W24	W48
**W0**	–	*0.997 (<10^−6^)*	*0.969 (<10^−6^)*	*0.986 <10^−6^)*	*0.794 (<10^−6^)*	*NA*
**W2**	**0.553 (<10^−6^)**	–	*0.985 (<10^−6^)*	*0.077 (<10^−6^)*	*0.681 (<10^−6^)*	*NA*
**W4**	**0.270 (<10^−6^)**	**0.654 (<10^−6^)**	–	*0.972 (<10^−6^)*	*0.564 (<10^−6^)*	*NA*
**W12**	**0.127 (<10^−6^)**	**0.316 (<10^−6^)**	**0.350 (<10^−6^)**	–	*0.763 (<10^−6^)*	*NA*
**W24**	**NA**	**NA**	**NA**	**NA**	–	*NA*
**W48**	**NA**	**NA**	**NA**	**NA**	**NA**	–

The longitudinal comparisons of patient 1 are indicated in the upper-right hemi-matrix of the table (*italics*); those of patient 2 are shown in the lower-left hemi-matrix of the table (**bold**).

**Table 4 ppat-1002314-t004:** Temporal population structure: Slatkin-Maddison test for comparison between longitudinal episomal HIV-1 DNA.

	Slatkin-Maddison test - Episomal HIV-1 DNA					
	Migrations (Probability)					
	W0	W2	W4	W12	W24	W48
**W0**	–	*1/4 (0.006)*	*5/5 (0.698)*	*6/6 (0.810)*	*2/4 (0.028)*	*NA*
**W2**	**1/3 (0.025)**	–	*1/4 (0.008)*	*1/4 (0.001)*	*1/3 (0.015)*	*NA*
**W4**	**1/3 (0.017)**	**1/2 (0.097)**	–	*6/7 (0.999)*	*3/4 (0.231)*	*NA*
**W12**	**NA**	**NA**	**NA**	–	*4/5 (0.496)*	*NA*
**W24**	**1/3 (0.018)**	**1/2 (0.099)**	**1/2 (0.099)**	**NA**	–	*NA*
**W48**	**3/4 (0.445)**	**1/3 (0.058)**	**1/3 (0.058)**	**NA**	**1/3 (0.029)**	–

The longitudinal comparisons of patient 1 are indicated in the upper-right hemi-matrix of the table (*italics*); those of patient 2 are shown in the lower-left hemi-matrix of the table (**bold**).

**Table 5:** Temporal population structure: Slatkin-Maddison test for comparison between longitudinal integrated HIV-1 DNA.

	Slatkin-Maddison test - Integrated HIV-1 DNA					
	Migrations (Probability)					
	W0	W2	W4	W12	W24	W48
**W0**	–	*1/2 (0.204)*	*1/2 (0.098)*	*1/2 (0.137)*	*1/2 (0.046)*	*NA*
**W2**	**3/4 (0.360)**	–	*1/3 (0.034)*	*2/3 (0.425)*	*3/3 (0.999)*	*NA*
**W4**	**3/4 (0.362)**	**1/3 (0.029)**	–	*1/3 (0.014)*	*2/4 (0.027)*	*NA*
**W12**	**6/7 (0.909)**	**3/4 (0.999)**	**3/4 (0.333)**	–	*3/4 (0.374)*	*NA*
**W24**	**NA**	**NA**	**NA**	**NA**	–	*NA*
**W48**	**NA**	**NA**	**NA**	**NA**	**NA**	–

The longitudinal comparisons of patient 1 are indicated in the upper-right hemi-matrix of the table (*italics*); those of patient 2 are shown in the lower-left hemi-matrix of the table (**bold**).

Our results revealed that in both episomal and integrated HIV-1 DNA samples, distinct genetic populations appeared at different time-points, suggesting that the appearance of each viral DNA form in blood could be the result of stochastic mobilization of different HIV-infected cells. This effect has been observed for residual viremia [12] and for different populations of CD4^+^ T-cells [7]. Furthermore, we did not observe any signs of evolution across longitudinal samples within episomal DNA or within integrated viral forms (Fig. 3 and 4). We found temporal variation but no evidence of continued evolution. Of note, patient 1's viruses harbor the mutation M184I in the reverse transcriptase of the integrated HIV sequences from weeks 0 and 2 (but not from weeks 4 or 24), which is associated to resistance to lamivudine and emtricitabine. This patient was under a regimen containing tenofovir, lamivudine, lopinavir, ritonavir and raltegravir. In contrast episomal sequences from weeks 0, 2 and 4 were wild type, which suggest that 2LTR circles were generated in a different cellular/anatomical compartment that is possibly less accessible to lamivudine.

### Different proportions of clonal sequences, rather than different sequences, determine population structure in patients under suppressive HAART

A population structure can occur at two levels: (i) different composition at the sequence level and (ii) different proportions of specific haplotypes. Therefore, the percentage of each clonal sequence of each DNA sample was represented (Fig. 3c–d and Fig. 4c–d). The results show that some haplotypes were shared among and within HIV-1 viral DNA forms, but unique haplotypes were also found. Moreover, when sequences were shared, the percentage of each haplotype was different between samples. This observation, together with the low molecular variability found in each sample (Table 1), suggests that patients under suppressive HAART have a limited variability in viral DNA sequences and that the presence and relative proportion of each clonal sequence might determine whether a population structure exists.

## Discussion

Previous reports have shown evidence of compartmentalization between residual plasma viremia and proviruses in fractionated and unfractionated PBMC [12]. However, this is the first time that episomal cDNA and integrated HIV-1 DNA genomes have been extensively compared. We found a statistically significant compartmentalization between episomal and integrated DNA samples in PBMC suggesting that, as with residual plasma viral RNA, episomal HIV-1 DNA forms are genetically distinct from proviral genomes and that they encompass two different genetic populations. In addition we have shown that in both episomal and integrated HIV-1 DNA samples, distinct genetic populations appeared, in a non-chronologic manner, at different time points. Longitudinal, non-chronological population structure between and within samples was detected in both circular episomal and proviral HIV-1 DNA viral forms. One explanation for our findings is that episomal and proviral sequences were generated in distinct cell types or anatomical compartments, possibly with different pharmacological penetration profiles. The detection of both DNA forms could result from stochastic mobilization from tissues to blood of a few infected cells, as their low molecular variance suggests. However, the labile nature of episomal DNA and its specific dynamics after raltegravir intensification [16] implies that the infection events that generated them occur in a pharmacologically privileged site (because the infections that generated them are occurring in the face of RT inhibitors) yet that is still accessible to raltegravir. A recent report shows that ileum may support ongoing productive infection in some patients on HAART, even if the contribution to plasma RNA is not discernible. In fact, raltegravir intensification contributed to a decrease in the cell-associated HIV RNA in this anatomic site relative to other gut sites or PBMC, suggesting that gut sites differ with respect to penetration by antiretroviral drugs, immunologic environments or the composition of CD4^+^ T cell populations [24]. Alternatively, our results might also suggest that cells containing these transiently-increased episomes might result from new infections with replication-competent viruses originating from rare proviruses not detectable in peripheral blood mononuclear cells, and that these episomes have had their integration blocked by raltegravir.

The lack of population structure or evolution in integrated HIV-1 genomes is best explained by the fact that proviruses are predominantly defective and archival. Although the provirus is the molecular precursor for all virions, only a very small percentage of proviruses are replication competent and only a small percentage of these would exist in a latent state- one capable of producing replication-competent virions. Therefore, temporal structure might simply be a result of continuous seeding of new cells over time. In this regard, multiple monotypic HIV-1 sequences have been observed across the uterine cervix and in blood, presumably as a result of the proliferation of cells harboring proviruses [25]. Although the origin of these cells with integrated HIV-1 DNA in our study remains unknown, forthcoming genotypic analysis across cell subpopulations in blood and tissues may cast light on this issue. Memory CD4^+^ T cells are thought to be a stable reservoir of HIV infection [26], [27], [28], [29], [30], [31]. The transient increases in 2LTR circles in PBMC from patients during early HAART in the absence of raltegravir, has recently been associated with the redistribution of 2LTR-enriched memory CD4+ T-cells from lymphoid tissues due to short-term decreases in immune activation [32]. In our study, changes in immune activation occurred more slowly. As such, distinct mechanisms appear to account for the changes in 2LTR circles. Interestingly, it has also recently been shown that the majority of a highly specialized subset of antigen-specific memory CD4^+^ T cells in mice were found to reside in a resting state in the bone marrow, surviving in close proximity to IL-7-secreting stromal cells [33]. Coincidently, IL-7 also induces homeostatic proliferation of human central memory CD4^+^ T cells without causing viral reactivation [34]. Therefore, the different population structure within integrated DNA, which is not coincident with the episomal DNA population, could be explained by the re-activation and mobilization of memory CD4^+^ T-cells from different niches in bone marrow without subsequent viral production, and could be a consequence of cellular rather than viral dynamics. Previous studies have examined rebounding viremia following treatment interruption and suggested that emerging viral variants result from the stochastic reactivation of different HIV-1 infected cells [4], [35], [36], [37]. Finally, infectious events during HAART can occur in multiple, temporary, small and locally scattered bursts [38] consistent with our observed genotypic compartmentalization. In addition, we observed that the cell-associated HIV proviral DNA remained relatively stable (Fig. S1) despite the large shifts in sequence populations observed during the study. This would not only mean that newly infected cell populations had undergone expansion but that other populations contracted at the same time. It is tempting to speculate that these dynamics might be consistent with the continuous trafficking between blood and tissues (preferentially lymphoid tissue) of stochastically activated antigen-specific memory CD4+ T-cells. We believe that it is unlikely that the results obtained reflect limited sampling, because we detected different proportions of shared haplotypes at different longitudinal time points (Fig. 1c,d and Fig. 2c,d). Sequence diversity restrictions due to sampling limitations would be reflected by either a completely different population structure or by invariable shared haplotypes in different samples. Moreover, at least in some time points, there is quite a large number of unique haplotypes (>1%), suggesting good depth.

Proviral HIV sequences are currently thought to be representative of archival HIV infection in an infected patient. Based on this hypothesis, sources of residual viremia other than CD4^+^ T-cells have been postulated as long-lived viral reservoirs [3]. Our observation that longitudinal detection of proviral genomes is dynamic in patients on HAART is important because it points to some limitations in the conclusions drawn from cross-sectional studies comparing HIV sequences in plasma and circulating T-cells.

Our results collectively suggest the presence of a chronic viral reservoir in which there is stochastic release of infectious virus and in which there are limited rounds of de novo infection. This could be explained by the existence of a limited cellular/anatomic reservoir in which de novo infection continues during HAART because some antiretroviral drugs do not effectively inhibit replication in this compartment. However, evidence that episomes transiently increase after raltegravir intensification suggests that this cellular/anatomic reservoir may be accessible to raltegravir, in contrast to other drugs. If proven in future work, the concept that ongoing replication during successful HAART originates from proviruses that are not detectable in peripheral blood mononuclear cells has important implications for the design of strategies aimed at viral eradication or functional cure. It indicates the need to further define the limited and covert cellular/anatomic reservoir in which ongoing HIV replication may occur during suppressive HAART.

## Materials and Methods

### Ethics statement

The study was approved by the Germans Trias i Pujol hospital review board and informed consent was obtained in writing from study participants.

### Study patients

We extensively analyzed longitudinal samples from 2 HIV-infected patients whose plasma viral load had been suppressed to <50 HIV-1 RNA copies/ml for 2 years on a stable HAART regimen. Both patients had participated in a previously reported raltegravir-intensification study [16], [17] were intensification of a three-drug suppressive HAART regimen resulted in a specific and transient increase in episomal DNA in a large percentage of patients. The original study was designed to compare populations of episomal and integrated HIV-1 DNA and plasma viral RNA in 5 patients with detectable episomal DNA before raltegravir intensification. Although plasma viral load assays employed 7 ml of plasma, we were unable to amplify viral RNA sequences for the majority of time points nor amplify episomal and integrated HIV-1 DNA from the majority of longitudinal samples in 3 patients. For this reason, structure comparisons between episomes and integrated HIV-1 DNA were only possible for the 2 subjects shown in this study. Episome and proviral DNA dynamics for both patients are shown in Fig. S1. HAART regimens included lopinavir, ritonavir, lamivudine, tenofovir and raltegravir for patient 1 and efavirenz, emtricitabine, tenofovir and raltegravir for patient 2. None of the included patients had previously been exposed to integrase inhibitors. Peripheral blood mononuclear cells (PBMC) and plasma samples included in this study encompassed weeks 0, 2, 4, 12, 24 and 48 after raltegravir intensification.

### Nucleic acid purification

A median of 6×10^7^ PBMC were obtained at weeks 0, 2, 4, 12, 24 and 48 after intensification and purified by Ficoll centrifugation and resuspended in 350 µl of P1 buffer (Qiaprep miniprep kit, Qiagen). 250 µl of cell suspensions were used for extrachromosomal HIV-1 DNA extraction (Qiaprep miniprep kit, Qiagen) using a modification for the isolation of low-copy-number plasmids. Total cellular DNA was purified from 100 µl of cell resuspension with a standard protocol (QIAamp DNA Blood Kit, Qiagen) as previously described [16].

### Amplification of integrated and episomal HIV-1 DNA

Analysis of HIV genomes from a sample containing a low copy number of HIV, such as PBMC from patients with undetectable viral load, can result in a high probability of resampling. The probability of resampling is related to both the number of target molecules during the amplification step and the number of sequenced clones. Therefore the higher the input of target molecules in the PCR and the higher the number of sequenced clones, the less likely the probability of resampling [39]. In order to avoid resampling, we extracted episomal and integrated DNA from a median of 6×10^7^ PBMC to increase the number of input molecules during the first PCR. In addition, only samples with individual clonal sequences higher than 1,500 after deep sequencing were considered for further analysis.

We used a two-step PCR to amplify the RT region of episomal and integrated HIV-1 DNA. Primers *Aluf* and *LA7* for integrated DNA and *Jct f* and *LA7* for episomal DNA were used as previously described [10]. Nested PCR amplification of the RT region (codons 150 to 250) was performed as part of the DS protocol (see below). Nested PCR of background controls with primers *DR pol f* and *DR pol r* [10], in parallel to 454 amplification, was carried out to ensure that nested PCR was specific for integrated and episomal DNA viral forms.

### Deep HIV-1 sequencing

Pooled, purified PCR products were used as template to generate a single amplicon covering codons 150 to 250 from the RT region. The amplicon library was generated in triplicate during 20 cycles of PCR amplification (Platinum Taq DNA Polymerase High Fidelity, Invitrogen, Carlsbad, CA) followed by pooling and purification of triplicate PCR products using magnetic beads (Agencourt AMPure Kit (Beckman Coulter, Benried, Germany) to eliminate primer-dimers. The number of molecules was quantified by fluorometry (Quant-iT PicoGreen dsDNA assay kit, Invitrogen, Carlsbad, CA). The quality of each amplicon was analyzed by spectrometry using a BioAnalyzer (Agilent Technologies Inc., Santa Clara, CA). Deep Sequencing (DS) was performed in-house on a 454 Life Science/Roche platform. The error rate of the *in-house* DS technique, as inspected with 992 pNL43 clonal sequences obtained with DS under the same conditions as those used for patient samples, was 0.07% (0.13%), which is close to previous reports [40]. This mismatch rate corresponds to a variability rate of 1.69×10^-5^, within the range of expected PCR error. The 99th percentile of mismatches would establish the threshold for nucleotide errors in 0.61%. Therefore, we decided to include for further analysis only patient clonal sequences present at ≥1% of the viral population.

### Sequence analysis

454 DNA amplicon sequences were aligned with an HXB2 reference sequence using Muscle v3.7 [41] and an independent alignment for each DNA, time-point and patient was built. In order to increase the number of sequences for further analysis and to avoid sequencing errors produced at the end of the sequencing run, we extracted from codon 50 to 209 from each of the sequences obtained with DS. Technical errors of the DS technique drive the introduction of indeterminations (introducing N instead of A, T, G or C) into the sequences. These indetermination were substitute by gaps. An *in-house* method was used to merge clonal sequences into unique sequences. Only clonal sequences present at ≥1% of the clonal population were used. Alignments are available upon request.

### Phylogenetic analysis

jModeltest v0.1.1 [42] was used to infer the best phylogenetic model to explain the alignment sequence evolution. This program is able to implement a discrete gamma distribution (Γ) which models the heterogeneity rate among sites. A neighbour-joining approach, as implemented in MEGA4 [18], was used to construct a phylogenetic tree with the best evolutionary model found in jModeltest v0.1.1.

### Population structure analysis

In order to detect differences in sequence composition between episomal and integrated DNA at different time-points, we performed two analyses, (i) an analysis of molecular variance (AMOVA) as implemented in the Arlequin software package [21], which is based on pairwise genetic distances and percentage of presence of each clone at each population, and (ii) a tree based topology method, the Slatkin-Maddison test [23] as implemented in HYPHY [43]. The Slatkin-Maddison tests involve estimating the number of migrations between populations, and determining whether the estimated number of migrations is less than expected if there were no compartmentalization. As the maximum possible number of migrations depends on the number of sequences analyzed from each compartment, a randomization test is performed to estimate a p value to assess the significance of compartmentalization. This approach has previously been used to study differences in sequence diversity between different HIV populations [12], [19], [20]. AMOVA is a genetic distance-based test where the frequency of a sequence variant (haplotype) *i* in the organ *j*, *x_ij_*, can be expressed as *x_ij_ = x + a_i_ + b_ij_*, where *a_i_* and *b_ij_* are episomal or integrated DNA and the haplotype within-DNA specific effects, respectively. These two factors have associated variances 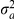 and 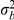 that can be described as total variance among haplotypes as 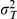. The *F_ST_* index measures the population differentiation. This value is defined as the ratio between 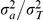, and it can be estimated from the usual partition of total variance into its components in a nested analysis of variance (ANOVA) [44]. We carried out AMOVA analysis by computing *F_ST_* with a distance matrix obtained from Arlequin program using the best evolutionary model found by jModeltest.

## Supporting Information

Figure S1 **Longitudinal dynamics of 2-LTR circles and total HIV-1 DNA during the study.**
(TIF)
