# Supplementary figures and images for: Deep Molecular Characterization of HIV-1 Dynamics under Suppressive HAART
Source: PLoS Pathog. 2011 Oct 27;7(10):e1002314. doi: 10.1371/journal.ppat.1002314 (PMC3203183; doi:10.1371/journal.ppat.1002314)

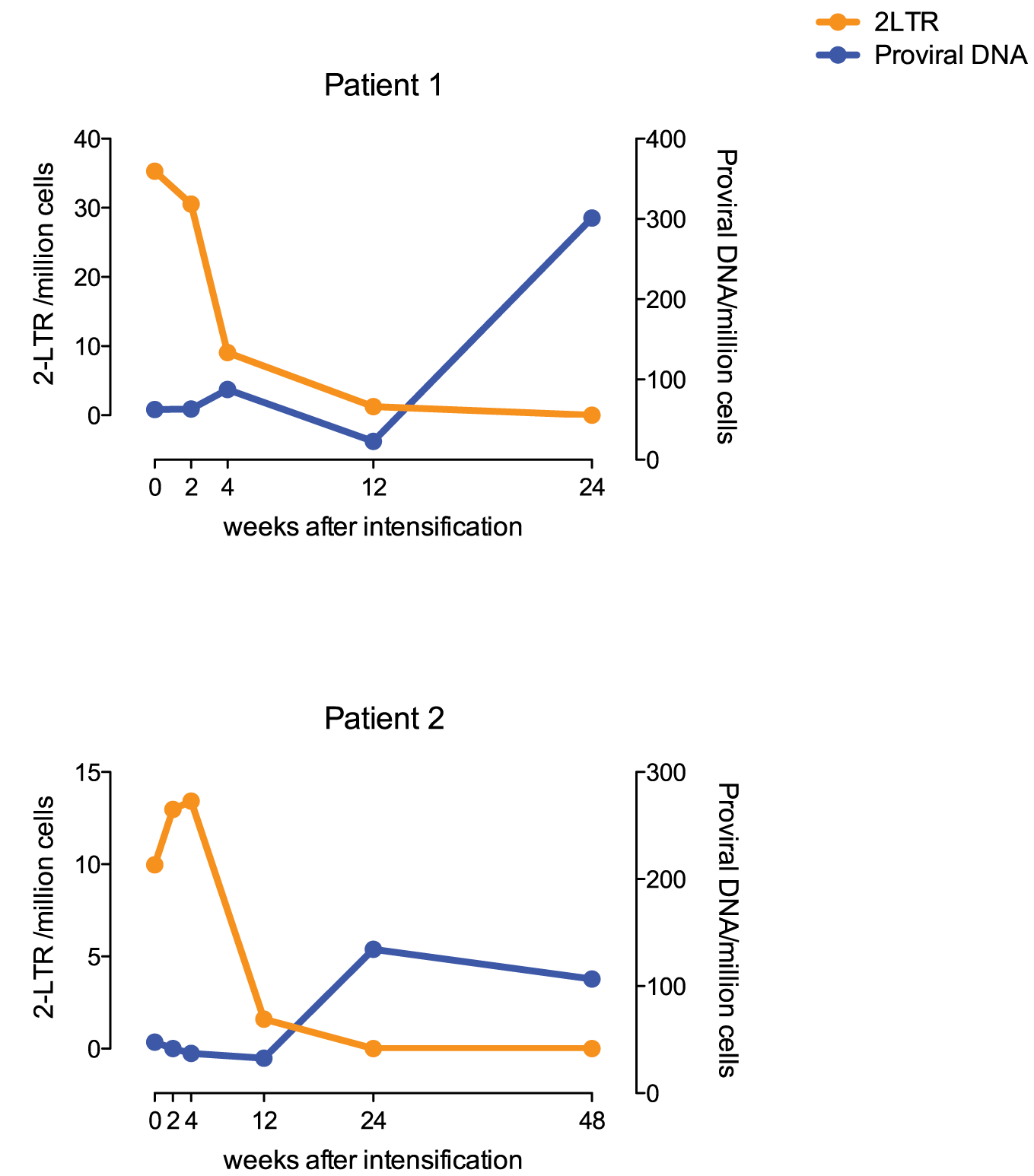

Supplement: Figure S1 — Longitudinal dynamics of 2-LTR circles and total HIV-1 DNA during the study. (TIF) [file ppat.1002314.s001.tif]
